# Dietary supplementation with hybrid palm oil alters liver function in the common Marmoset

**DOI:** 10.1038/s41598-018-21151-0

**Published:** 2018-02-09

**Authors:** Flavia Spreafico, Rafael Carvalho Sales, Judit Gil-Zamorano, Priscylla da Costa Medeiros, Maria-Jesús Latasa, Monique Ribeiro Lima, Sergio Augusto Lopes de Souza, Roberto Martin-Hernández, Diego Gómez-Coronado, Eduardo Iglesias-Gutierrez, Diana C. Mantilla-Escalante, Maria das Graças Tavares do Carmo, Alberto Dávalos

**Affiliations:** 1 Instituto de Nutrição Josué de Castro, Universidade Federal do Rio de Janeiro, Rio de Janeiro, Brazil; 2 Laboratory of Epigenetics of Lipid Metabolism, Instituto Madrileño de Estudios Avanzados (IMDEA)-Alimentación, CEI UAM+CSIC, Madrid, 28049 Spain; 3 Laboratório de Marcação de Células e Moléculas, Departamento de Radiologia, Faculdade de Medicina, Hospital Universitário Clementino Fraga Filho, UFRJ, Rio de Janeiro, Brazil; 4 Servicio de Bioquímica-Investigación Hospital Universitario Ramón y Cajal, IRYCIS, Madrid, 28034 Spain; 5 CIBER Fisiopatología de la Obesidad y Nutrición (CIBEROBN), Instituto de Salud Carlos III, Madrid, 28029 Spain; 6 Department of Functional Biology (Physiology), University of Oviedo, Oviedo, 33003 Spain; 7 Universidad Autónoma de Chile, Santiago, 7500912 Chile

## Abstract

Hybrid palm oil, which contains higher levels of oleic acid and lower saturated fatty acids in comparison with African palm oil, has been proposed to be somehow equivalent to extra virgin olive oil. However, the biological effects of its consumption are poorly described. Here we have explored the effects of its overconsumption on lipid metabolism in a non-human primate model, the common marmoset. Dietary supplementation of marmoset with hyperlipidic diet containing hybrid palm oil for 3 months did not modify plasma lipids levels, but increased glucose levels as compared to the supplementation with African palm oil. Liver volume was unexpectedly found to be more increased in marmosets consuming hybrid palm oil than in those consuming African palm oil. Hepatic total lipid content and circulating transaminases were dramatically increased in animals consuming hybrid palm oil, as well as an increased degree of fibrosis. Analysis of liver miRNAs showed a selective modulation of certain miRNAs by hybrid palm oil, some of which were predicted to target genes involved in cell adhesion molecules and peroxisomal pathways. Our data suggest that consumption of hybrid palm oil should be monitored carefully, as its overconsumption compared to that of African palm oil could involve important alterations to hepatic metabolism.

## Introduction

In the last decades, the increasing consumption of processed and industrialized food with low nutritional quality has correlated with an increment of chronic diseases prevalence, such as obesity and cardiovascular diseases^1,2^. After it has been proved that partially hydrogenated fat–which is rich in trans isomers– was harmful to health, its use was forbidden in different countries. African Palm oil (AP) (extracted from *Elaeis guineensis*, african palm tree) has been the vegetable oil most widely used by the food industry as a trans-fat substitute even though it is rich in saturated fatty acids. However, recently, Chen *et al*. showed that for each kilogram of palm oil consumed annually per capita, the mortality due to ischemic cardiomyopathy increases the equivalent to 68 for 100,000 deaths in developed countries^1^. Even if metabolic consequences of AP consumption are still controversial^3^, its consumption remains, a source of major concern within health policies regarding the reduction of cardiovascular and metabolic diseases risk.

Interspecific hybridization between African oil palm (*Elaeis guineensis)* and South American species, *Elaeis oleifera* has been exploited, with the objective of developing varieties as productive as African oil palm, together with resistance to diseases and pest, reduced height and improved nutritional value, specifically, increased monounsaturated oleic acid and reduced saturated fatty acids levels^4,5^. EMBRAPA (Empresa Brasileira de Pesquisa Agropecuária, Brazilian Corporation of Agricultural Research) has developed the first Brazilian interspecific hybrid cultivar between *Elaeis oleifera* and *Elaeis guineensis*, which has all these characteristics, i.e. high productivity and improved nutritional value^6^. In this context, hybrid palm oil (HP) has been proposed as a promising new lipid source for industrialized products and a substitute for AP that could bring benefits to human health.

Previous studies on the chemical composition of HP suggest that the ripening status influences the amount of antioxidant phenolic content in the fruit^7^. Regarding its fatty acid profile, Mozzon *et al*., suggested that hybridization seemed to modify the biosynthesis of fatty acids rather than their assembly in the triacylglycerols^8^. In fact, they found that the *sn*-2 position of triacylglycerols in HP was mainly esterified with oleic acid (≈10% increment compared to that of AP)^8^. This apparent healthier profile of fatty acids composition of HP was tested and compared to that of extra virgin olive oil (EVOO) in dietary supplementation^9,10^. When consumed for three months, both oils increased the degree of unsaturation of erythrocyte membrane lipids^9^. Moreover, dietary supplementation with HP showed similar effects on plasma lipids composition compared to that of EVOO (no differences between both groups)^10^. Researchers have suggested that the observed beneficial effects of HP consumption support the concept of HP as a “tropical equivalent to olive oil”.

On the other hand, recent studies suggest that there is no clear evidence to conclude that dietary saturated fat is associated with an increased risk of CVD^11–14^. Furthermore, the metabolic consequences of AP consumption are still controversial. Its effects can be favorable or unfavorable when compared with other types of fat sources, according to the evaluated parameters^3^. According to Wilson *et al*.^15^, these controversial metabolic effects of AP can be justified by the high presence of tocopherols and tocotrienols in their fat. These tocols can promote an antithrombotic state by reducing platelet aggregation and modulating prostanoid synthesis. But once the fat is refined, bleached and deodorized, depending on the degree of processing, the antithrombotic properties are inhibited^15^. Moreover, although no detrimental side effects of monounsaturated fatty acids (MUFA) rich diets were reported, there is still no consensus regarding monounsaturated fatty acids consumption recommendations^16^. Indeed, some large studies also suggest that substitution of saturated fatty acids by MUFAs can increase the risk of ischemic heart disease^13^. In this context, whether the consumption of HP–enriched in MUFAs–should be promoted due to its putative beneficial effects is still unclear.

miRNAs are posttranscriptional regulators of gene expression. Increasing evidence suggests that miRNAs can be modulated by diet^17^. Indeed, fatty acids have been shown to influence miRNAs expression in different metabolic tissues including intestine^18^, adipose tissue^19^ and liver^20^. Whether saturated or MUFAs influence liver miRNA expression is poorly characterized^21^. Moreover, whether HP can influence miRNA expression is completely unknown.

**Figure 1:**
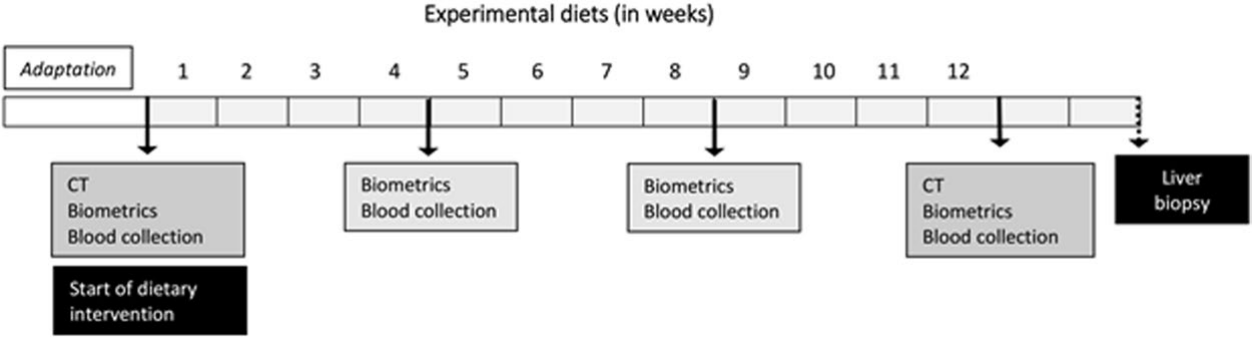
Experimental design.

The main objective of this study was to evaluate the effects of overconsumption of HP, source of unsaturated fatty acids–obtained from the interspecific hybrid grown in EMPRAPA/Brazil–, *vs*. that of AP, rich in saturated fatty acids on lipid metabolism, and to identify liver miRNAs that could be modulated by these dietary fatty acids. For this purpose, we used the common marmosets, a New World primate species that has great phylogenetic similarity with humans, being omnivorous animals, with diversified eating habits and easy adaptation to experimental diets. Nonhuman primates have also a lipid metabolism that is similar to ours and are recognized as a good animal model for dyslipidemia and metabolic disorders studies^22^. The experimental design is shown at Fig. 1.

## Results

### Fatty acid profile of experimental diets

First, we decided to study the detailed lipid composition of the experimental diets (Table 1) in order to assess their differences. The fatty acid profile of the experimental diets is shown in Table 2 and it is compatible with the diets formulation, since 2% of soybean oil was added to prevent possible deficiencies in essential fatty acids. Even though, the original fatty acid profile of the fats was maintained in the diets (Table 1). African palm oil diet (AP) presented 6% more saturated fatty acids, mainly because of palmitic acid content, and the HP diet (HP) showed 4% higher amounts of MUFAs, mainly due to oleic acid content.

**Table 1:** Experimental diets composition shown as g/100 g.

Ingredients	g/100 g
Ground Corn	8.80
Wheat middlings	8.50
Rice bran	8.00
Sucrose	19.50
Dehulled soybean meal	5.00
Casein	7.00
Powder milk	7.00
Albumin	11.20
Chicken meal	2.90
Soybean oil	2.00
Palm oil*	14.30
Fibre	2.00
Vitamins and minerals mix	3.86
Butylhydroxytoluene	0.02
***Kcal/100 g***	***422.08***
*Protein*	*16%*
*Carbohydrate*	*43%*
*Lipid*	*41%*

*African palm oil or hybrid palm oil.

**Table 2:** Characterization of diets according to their fatty acid profiles.

Fatty acids (%)	African Palm	Hybrid Palm
***Saturated FA***		
Palmitic (C16:0)	35.88 ± 0.02	30.68* ± 0.30
Stearic (C18:0)	5.55 ± 0.01	5.23 ± 0.02
∑ **SFA**	**45.25 ± 0.13**	**39.20 ± 0.31**
***Monounsaturated FA***		
Oleic (C18:1 9c)	37.11 ± 0.01	41.64* ± 0.15
∑ **MUFA**	**43.90 ± 0.01**	**47.72 ± 0.34**
***Poliunsaturated FA***		
LA (C18:2 9c 12c)	9.83 ± 0.16	11.23 ± 0.40
AA (C20:4 5c, 8c, 11c, 14c)	0.08 ± 0.00	0.08 ± 0.00
∑ **n-6 FA**	**10.11 ± 0.26**	**11.65 ± 0.21**
AAL (C18:3 9c 12c 15c)	0.54 ± 0.03	1.04 ± 0.01
EPA (C20:5 5c, 8c, 11c, 14c, 17c)	0.11 ± 0.00	0.12 ± 0.00
DHA (C22:6 4c, 7c, 10c, 13c, 16c, 19c)	0.03 ± 0.01	0.01 ± 0.00
∑ **n-3 FA**	**0.69 ± 0.05**	**1.25 ± 0.08**
∑ **PUFA**	**10.82 ± 0.31**	**12.93 ± 0.13**
**Saturated/monounsaturated FA ratio**	**1.03**	**0.82**
**Saturated/poliunsaturated FA ratio**	**4.18**	**3.03**

AP: African Palm Oil; HP: Hybrid Palm Oil. Σ SFA: sum of saturated fatty acids; Σ MUFA: sum of monounsaturated fatty acids; Σ PUFA: sum of polyunsaturated fatty acids; LA: linoleic acid; ALA: alfa linolenic acid; Σ n-6: sum of n-6 polyunsaturated fatty acids; Σ n-3: sum of n-3 fatty acids. AA: Arachidonic Acid; EPA: Eicosapentaenoic Acid; DHA: docosahexaenoic acid. Values are expressed as mean ± standard error (3 samples of each diet). *Means statistical difference at p < 0.05 HP vs. AP.

### Body weight evolution

Next, we decided to determine whether there were differences on food intake due to the experimental diets. Animals consumed normally the prepared diets during the intervention period, both during normocaloric and hyperlipidic phases. No apparent differences in food intake were observed. Despite the consumption of hyperlipidic diets, no significant changes in body weight during the nutritional intervention were observed, either when analyzed by sex or computing both sexes together (Supplementary Figure S1).

### Biochemical parameters

**Figure 2:**
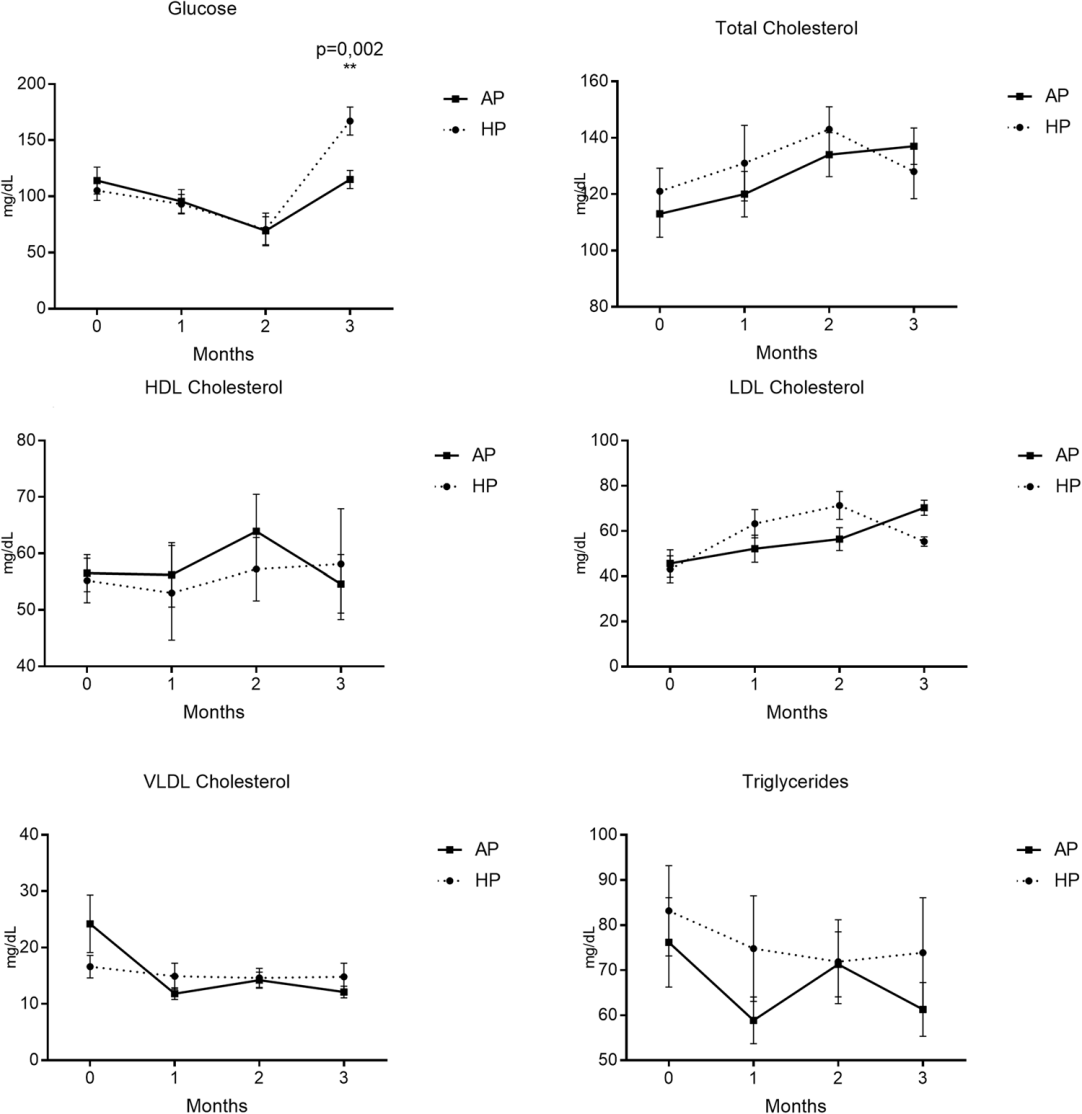
Biochemical parameters analyzed during the nutritional intervention (n = 10 animals per group). AP: African Palm Group; HP: Hybrid Palm Group. Glucose, Total cholesterol, HDL cholesterol, LDL cholesterol, VLDL cholesterol, and triglycerides were determined with standard colorimetric methods. Values expressed as mean ± SEM. **Indicates a statistical difference at p < 0.02.

**Figure 3:**
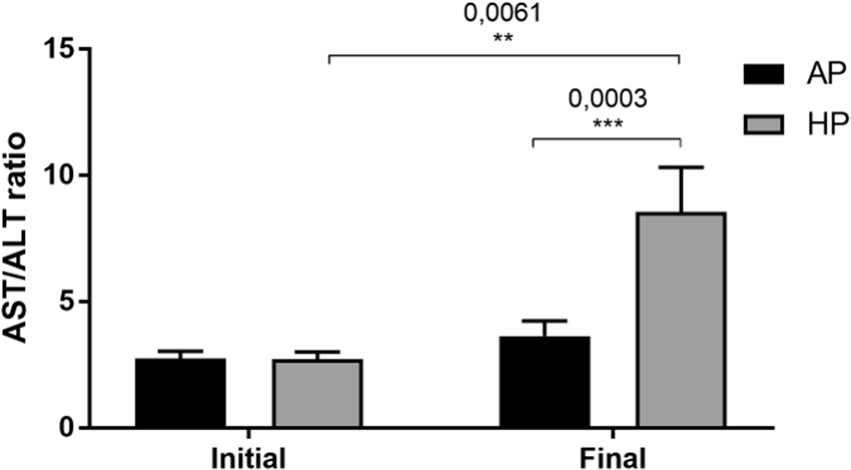
AST/ALT Ratio at the beginning and at the end of the study. (n = 9 animals per group). AP: African Palm Group; HP: Hybrid Palm Group.

Since we did not observe any apparent changes in body weight, we decided to determine whether the experimental diets had any effect on metabolism. Blood biochemical parameters, including cholesterol and its fractions, glucose and liver function markers were analyzed (Fig. 2). Dietary ingestion of the experimental oils did not elicit any significant changes in lipid levels (Fig. 2). This was further confirmed when lipoprotein profiles were analyzed by FPLC (Supplementary Figure S2). However, glucose levels were significantly changed by the consumption of both hyperlipidic diets, showing decreasing levels for the first two months of nutritional intervention and then surprisingly increasing glucose levels on the last month, with a greater increment in HP group (p = 0.002) when compared to AP group (Fig. 2). Also the ratio AST/ALT was calculated as a marker for hepatocellular lesion^23^. As shown in Fig. 3, both groups had an increase in this parameter, however HP group showed significant higher values.

### Liver volume analysis

**Figure 4:**
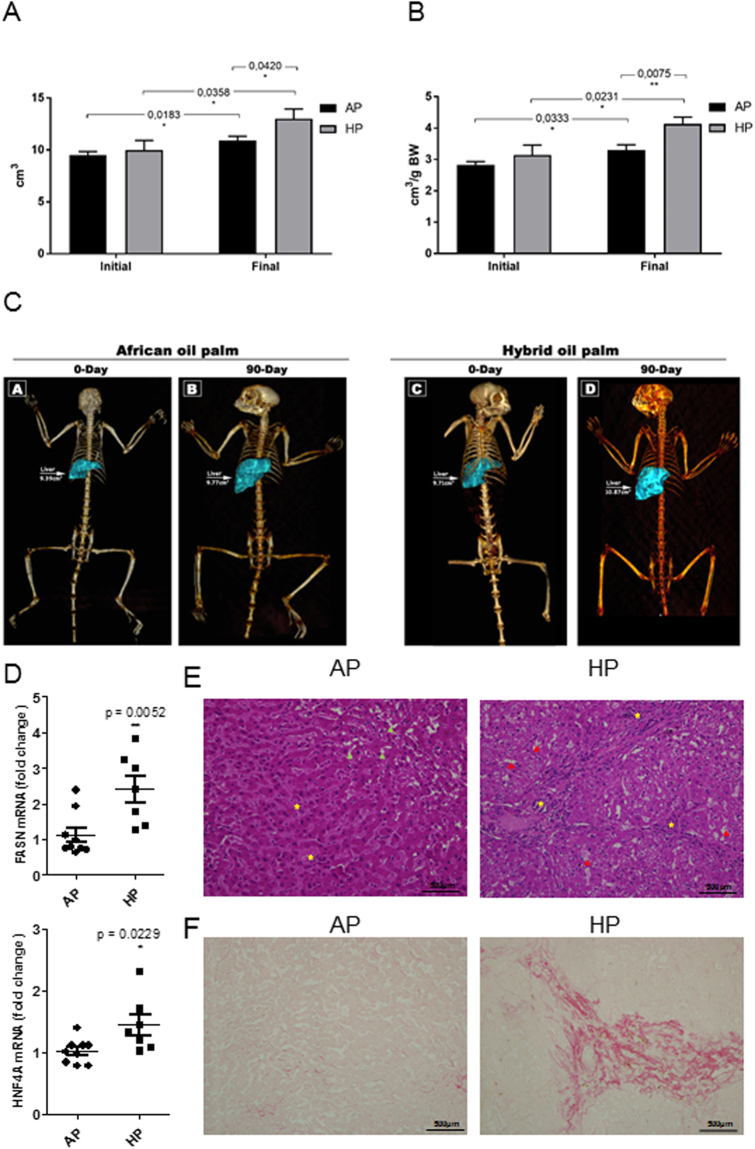
Hepatic volume of experimental animals at the beginning and at the end of the study. (n = 10 animals per group). AP: African Palm Group; HP: Hybrid Palm Group. (**A**) Liver volume expressed in cm^3^. (**B**) Liver volume corrected by body weight. Values are expressed as mean ± standard error. *Indicates statistical significance at p < 0.05 and ** at p < 0.02. (**C**) Scanner images of marmosets fed with either AP or HP diet (representative images), both at the beginning and at the end of the nutritional intervention. Images were obtained by PET/CT Scan. (**D**) Gene expression analysis of fatty acid synthase (FASN) and hepatic nuclear factor 4 alpha (HNF4A) in liver samples of animals fed with AP or HP. n ≥ 7 per group. (**E**) Liver histological cross-sections from marmoset fed either AP or HP (Hematoxylin-eosin). Green arrowhead: microvesicular steatosis; red arrowhead: steatosis; yellow stars: inflammatory infiltrate. (**F**) Photomicrograph of cells with collagen staining (Sirius red). Histological analysis are representative of n = 3 animals per group.

The lack of major changes in body weight of the experimental animals prompted us to evaluate other parameters that may counterbalance this apparent lack of effects. Therefore, we decided to measure the liver volume as a surrogate of the assessment of the interference of experimental diets on animal’s metabolism. Liver volume was analysed by CT. As expected due to the consumption of hyperlipidic diets, liver volume of non-human primates consuming both diets elicited an abnormal increase. Figure 4 depicts the results of hepatic volume of the experimental animals. Both groups presented hepatic volume increment, even when values are corrected by body weight (Fig. 4B). The two experimental groups showed an increase in hepatic volume between the beginning and the end of the experimental diets. The AP group presented a 15% increase in hepatic volume, whereas, surprisingly, the HP group had a 30% increase, that is, twice that of the AP fed (Fig. 4A,B). When corrected for body mass, the values are slightly higher, maintaining the ratio of 16% increase for AP and 31% for HP. However, while at the beginning of the experiment both groups had similar hepatic volume, by the end of it, the HP diet group presented a hepatic volume 19% higher than the AP group and, when corrected for body weight, this difference increased to 25%. Lipid determination of liver tissues at the end of the experiment showed that AP had 3.08 ± 0.12% fat, while HP had ≈3-fold higher (9.84 ± 0.67%; *P* = 0.0001). Tissue analysis of the fatty acid synthase (FASN) gene showed an increased expression of the mRNA in animals consuming HP (Fig. 4D). While the hepatic nuclear factor 4 alpha (HNF4A), a gene involved in hepatic reparation process, also increased its mRNA expression by HP supplementation. Histological analysis showed an increase number of steatotic cells and inflammatory infiltrate in animals consuming HP (Fig. 4E). Moreover, collagen staining (Fig. 4F) showed an increased degree of fibrosis in animals fed HP compared to that of AP.

### Fatty acid composition of total lipids in liver tissues

To elicit the cause for the greater effect of HP diet on liver volume and fat content increase, we determined the fatty acid profile of liver tissue. As shown in Table 3, the differences between both groups for some fatty acids are very subtle and only two analysis showed statistical differences. The net changes, though, reflect a liver lipid composition poorer on unsaturated fatty acids when compared with the diet lipid composition. This is shown both at the ∑ PUFA (increased in the HP diet vs. the AP diet but decreased in the HP fed liver vs. the AP fed liver) and the saturated/polyunsaturated FA ratio (decreased in the HP diet vs. the AP diet but slightly increased in the HP fed liver vs. the AP fed liver).

**Table 3:** Fatty acid profile of liver tissue from the two experimental groups.

Fatty acids (%)	African Palm	Hibrid Palm
***Saturated FA***		
Palmitic (C16:0)	19.15 ± 0.92	17.78 ± 0.33
Stearic (C18:0)	12.04 ± 1.27	13.24 ± 1.33
∑ **SFA**	**32.66 ± 1.10**	**33.07 ± 1.64**
***Monounsaturated FA***		
Palmitoleic (C16:1 9c)	1.01 ± 0.05	1.54* ± 0.06
Oleic (C18:1 9c)	20.26 ± 1.41	22.36 ± 0.50
∑ **MUFA**	**22.23 ± 1.45**	**24.61 ± 0.44**
***Poliunsaturated FA***		
LA (C18:2 9c 12c)	22.11 ± 0.96	19.48 ± 1.29
AA (C20:4 5c, 8c, 11c, 14c)	9.75 ± 1.19	8.51 ± 1.57
∑ **n-6 FA**	**37.85 ± 2.19**	**35.89 ± 2.30**
AAL (C18:3 9c 12c 15c)	0.89 ± 0.09	0.94 ± 0.05
EPA (C20:5 5c, 8c, 11c, 14c, 17c)	0.10 ± 0.02	0.16 ± 0.03
DHA (C22:6 4c, 7c, 10c, 13c, 16c, 19c)	4.02 ± 0.35	3.51 ± 0.34
∑ **n-3 FA**	**7.26 ± 0.48**	**6.30 ± 0.63**
∑ **PUFA**	**45.10 ± 2.12**	**42.20 ± 2.11**
**Saturated/monounsaturated FA ratio**	**1.49 ± 0.10**	**1.34 ± 0.05**
**Saturated/poliunsaturated FA ratio**	**0.73 ± 0.06**	**0.79 ± 0.08**
**16:1/16:0 ratio**	**0.05 ± 0.00**	0.09* ± 0.01
**18:1/18:0 ratio**	**1.76 ± 0.07**	**1.57 ± 0.08**

AP: African Palm Oil; HP: Hybrid Palm Oil. Σ SFA: sum of saturated fatty acids; Σ MUFA: sum of monounsaturated fatty acids; Σ PUFA: sum of polyunsaturated fatty acids; LA: linoleic acid; ALA: alfa linolenic acid; Σ n-6: sum of n-6 polyunsaturated fatty acids; Σ n-3: sum of n-3 fatty acids. AA: Arachidonic Acid; EPA: Eicosapentaenoic Acid; DHA: docosahexaenoic acid. Values are expressed as mean ± standard error (n = 8–10). ∗Means statistical difference at p < 0.05).

The 16:1/16:0 ratio showed significant higher levels in HP diet indicating either increased hepatic Stearoyl-CoA desaturase (SCD1) or higher availability of palmitic acid (C16:0) as a substrate for the enzymatic reaction (p < 0.0001), suggesting that the increased SCD1 activity in the liver of HP group might be involved in the process of hepatic lipid accumulation with potential to promote NAFLD (Table 3).

### Hybrid palm diet modifies hepatic miRNAs

**Figure 5:**
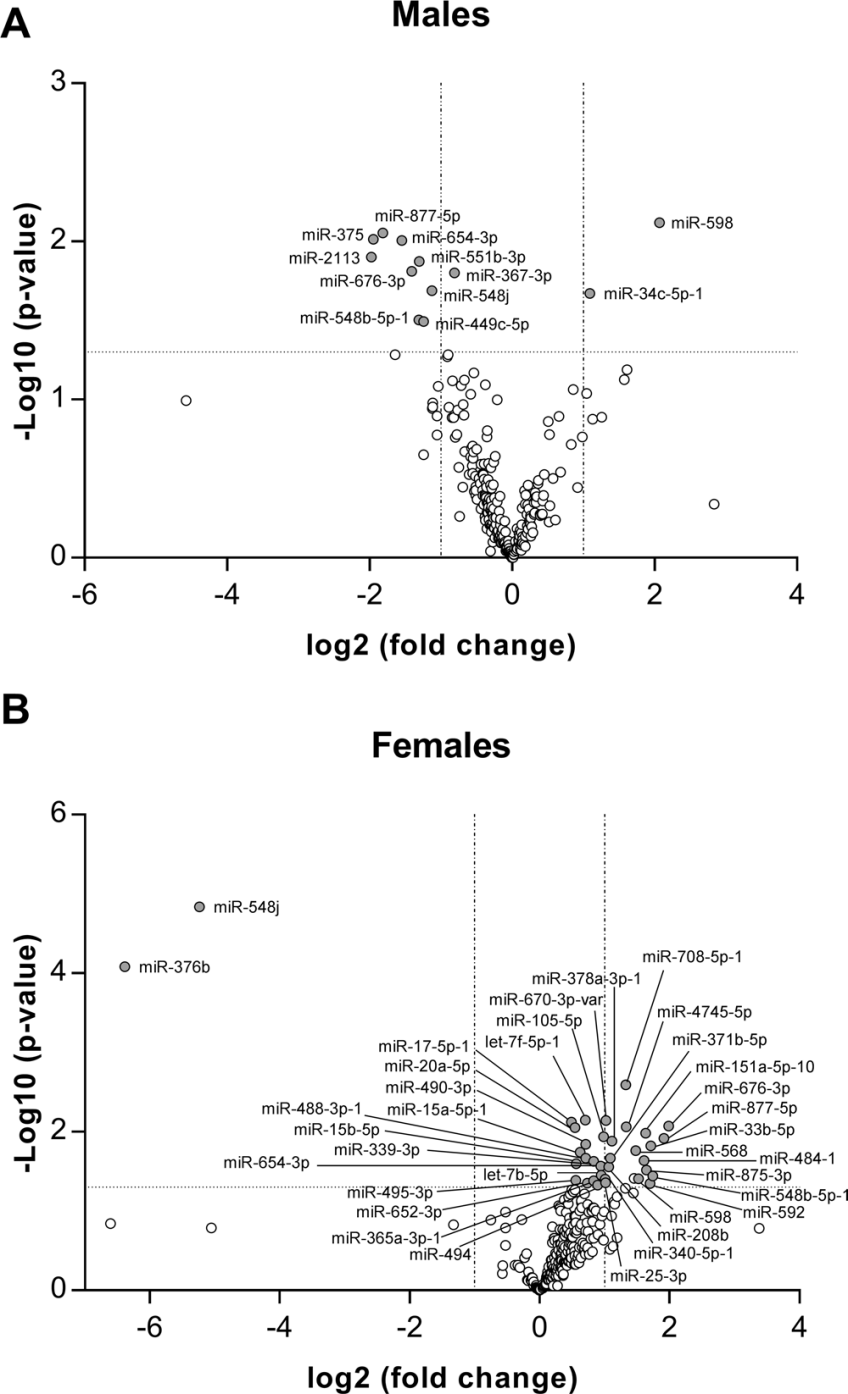
HP supplementation modulates miRNA expression. Volcano plots of hybrid palm oil-modulated miRNAs in marmoset liver. (**A**) Males; (**B**) Females. miRNA expression was analysed by RT-qPCR (n = 9 animals per group). Labelled miRNAs were selected for further validation.

**Figure 6:**
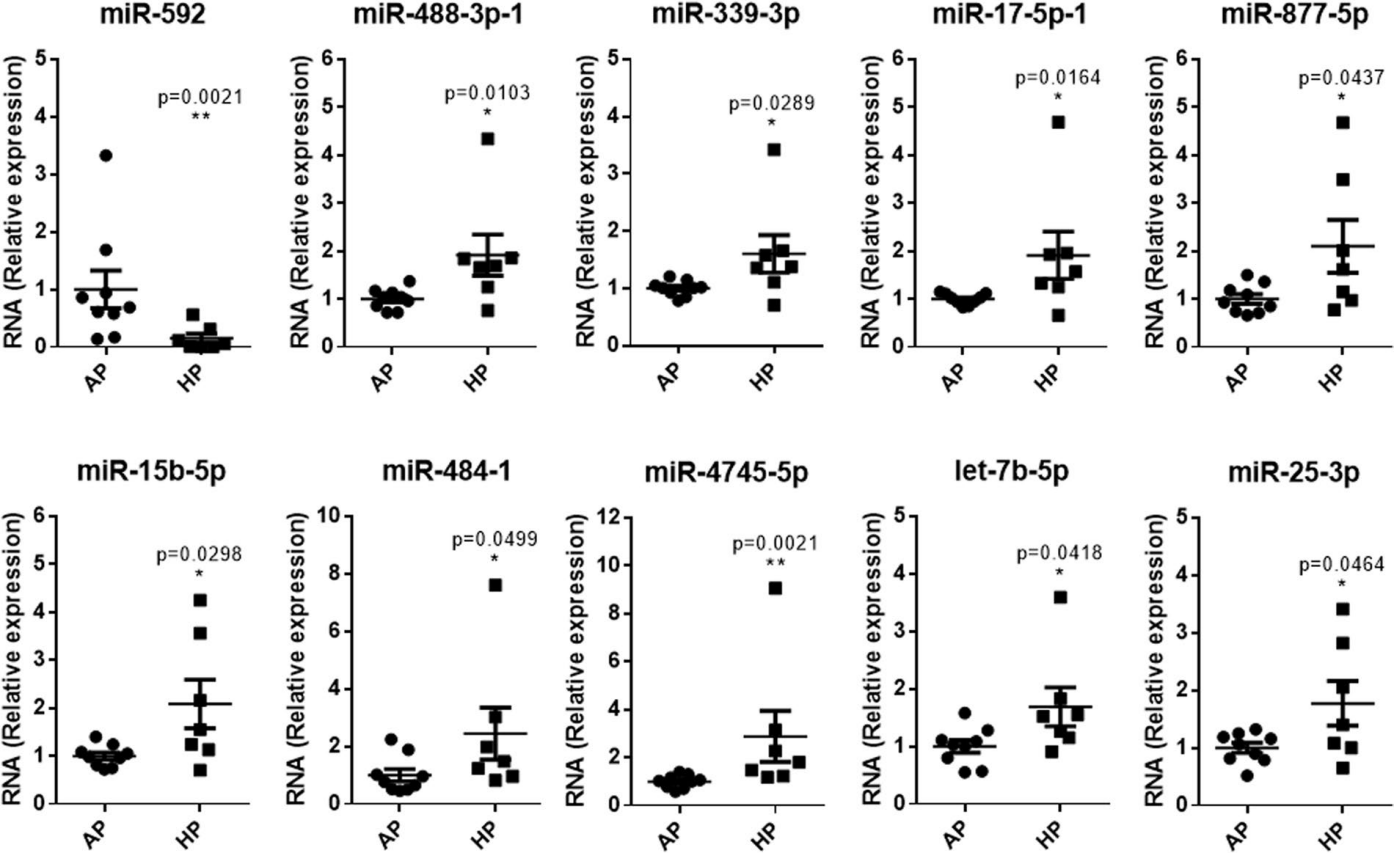
Liver miRNAs modulated by HP diet. Hepatic miRNAs validated in both sexes. AP: African Palm Group; HP: Hybrid Palm Group. miRNA expression was analysed by RT-qPCR (n = 9 animals per group). Values are expressed as mean ± standard error. *Indicates statistical significance at p < 0.05.

Hepatic lipid accumulation, such as it is observed in NAFLD, can be promoted by various causes, among them the upregulation of lipid metabolism related genes expression, leading to an increased hepatic de novo lipogenesis^24,25^. Epigenetic factors, such as DNA methylation, histone modifications or microRNAs (miRNAs), have been involved in liver disease^26,27^. Over the last decade, miRNAs have emerged as critical regulators of lipid metabolism (for a review, see Aryal *et al*.^28^ and herein references). In order to gain insight into possible molecular mechanisms modulated by HP that could be leading to the observed hepatic lipid accumulation, we next evaluated hepatic miRNAs. A selection of miRNAs from marmoset, which are conserved in humans (see Materials and Methods for details), was first screened in both male (Fig. 5A) and female liver samples (Fig. 5B) obtained from biopsies. A list of 45 miRNAs (sum of male and female miRNAs whose expression was found to be modulated) was next validated with a different set of primers by qRT-PCR. 10 miRNAs were consistently validated (Fig. 6) to be differentially expressed by HP diet, namely miR-592, miR-488-3p, miR-339-3p, miR-17-5p-1, miR-877-5p, miR-15b-5p, miR-484-1, miR-4745-5p, let-7b-5p and miR-25-3p. The rest of the selected miRNAs for validation were not differentially modulated when assessed in both sexes (Supplementary Figure S3). Gene ontology analysis of predicted targets of these miRNAs showed that neuron projection development (GO:0031175), or pathways related to nicotinic acetylcholine receptor signalling (Panther:P00044), cell adhesion molecules (KEGG:04514) or peroxisome (KEGG:04146) are among the possible targets of these miRNAs (Supplementary Table S1).

## Discussion

Dietary FAs play significant roles in the cause and prevention of cardiovascular disease. Palm oil may be an unhealthy fat, mainly extracted from *E. guineensis* (AP) because of its high saturated FA content, specifically palmitic acid (C16:0 ≈ 44%), and its use in foods and food products has been somewhat discouraged. On the other hand, although controversies persist concerning the association between the intake of dietary saturated fatty acids and CVD risk^29–31^, several studies have shown the beneficial role of diets with a relatively high MUFA content on CVD risk factors, obesity and diabetes. These beneficial MUFA are mainly provided by the Mediterranean diet and, specifically, by olive oil, oleic acid-rich oils^32^. In this context, higher degree of FA unsaturation is therefore a desirable characteristic to alter in palm oil. Hybrids between *E. guineensis* × *E. oleifera* excited much interest, because of the shorter size, more resistance to several diseases, higher desaturation of palm oil and, in particular, putative healthier oil, constituting an alternative approach to increase oleic acid content in palm oil^4,5^. Seeds of high yielding interspecific hybrids palm oils (HP) are produced in Brazil by EMBRAPA/Dendê do Pará S.A.12 (MANICORÉ hybrids), using wild *E. oleifera* palms indigenous to the Coari and Manicoré municipalities in the Amazon river basin.

In the present study, our results of fatty composition revealed significant differences between the HP and AP produced in Brazil, where HP presents a higher level of oleic acid (49.6 ± 0.01 *vs* 42.1 ± 0.01 in original oils, data not shown) together with a lower palmitic acid content (32.8 ± 0.1 *vs* 40.9 ± 0.2 in original oils, data not shown). Furthermore, as expected, a high-fat diet manufactured from the HP showed higher contents of monounsaturated and lower of saturated fatty acids compared to AP. However, the amount of the polyunsaturated or essential fatty acid, linoleic acid, does not differ significantly (Table 2). Comparing our results with those of Mozzon *et al*.^8^, the fatty acid composition of the HP grown in Colombia showed higher percentage of oleic acid (54.6% ± 1.0) and similar saturated fatty acid content (33.5% ± 0.5), while the percentage of others fatty acids does not undergo significant changes. Moreover, a recent positive effect of HP –grown in Colombia– on human plasma lipids has been observed, where after three months of 25 ml per day HP consumption, the total cholesterol levels decreased by 6.3%, whereas LDL-C concentration decreased by more than 13%^10^. This is the only study found in the literature that shows similar effects on plasma lipids composition compared to EVOO^10^. On the other hand, our results provide evidence that a high-fat diet richer in HP –grown in Brazil– does not result on a healthier liver lipid content profile in marmoset.

The increased liver volume of animals consuming HP diet prompted us to search for other markers of liver damage. The AST/ALT ratio is an index used in clinical practice as a non-invasive method of determining the progression of various liver diseases, especially NAFLD^23^. We found that HP diet dramatically increased this ratio, suggesting an increase in liver injury in this group, which is in accordance with disease progression observed in humans^33^. Excessive accumulation of lipids in the liver is also a characteristic of NAFLD, which leads to liver damage when inappropriately metabolized. Besides that, the hyperglycaemia founded in HP group (Fig. 2) at the end of the study, also indicates impairment in the liver function, once it is known that fat accumulation in the liver suppresses insulin signaling in hepatocytes, leading to hyperglycemia^34^. Our results also show a three-fold increase in lipid accumulation in the liver, without any apparent changes in plasma lipid levels or lipoprotein profile. Thus, some of the discrepancies between the present study and the literature can be explained by differences in important variables such as the reference or control group, which in the present study is the African palm group and/or the context of the diet which is a high-fat diet. Indeed, the major difference between both groups was just the different amount of oleic acid, which at that concentration (49.6% ± 0.01 *vs* 42.1% ± 0.01 in original oils, data not shown) might not induce appreciable changes in circulating lipids levels. In the context of a high fat diet, it has been previously described that some positive effects related to dietary fatty acids can be lost under high-fat intake^35^, such as in our study. Indeed, animal studies when fed high fat diet have shown that palmitic acid or palm oil enriched diet can induce obesity, hepatic steatosis or changes in the microbiota^36,37^. In humans, only one study shows that overfeeding SFAs from palm oil increases hepatic fat compared to that of n-6 PUFAs (sunflower oil)^38^. Thus, whether this HP grown in Colombia, when consumed in large quantities in the diet also produce liver damage in humans, as observed here for marmosets, has not been evaluated.

There is evidence that the metabolic effects of dietary TAG depend not only on their FA composition but also on the stereochemical configuration of specific FA in the TAG molecule. In Colombia, Mozzon *et al*., suggested that hybridization seemed to modify the biosynthesis of fatty acids rather than their assembly in the triacylglycerols^8^. In fact, they found that the *sn*-2 position of triacylglycerols in HP was mainly esterified with oleic acid (≈10% increment compared to that of AP)^8^. This apparent healthier profile of fatty acids composition of HP was tested and compared to that of EVOO in dietary supplementation^9,10^. Although more direct experiments are required in the HPs produced in Brazil, it is possible that the HP grown in Colombia, due to its higher proportion of unsaturated FA chains in position 2 of the TAG molecule^8^, could be quickly oxidized, resulting in a significant impact on the circulating lipid levels when in dietary supplementation.

The finding in this study of increased total lipid in liver tissue from animals consuming HP diet, motivated us to study the ratio of C18:1/C18:0 and C16:1/C16:0 and therefore, SCD1 activity was indirectly estimated. The enzyme SCD1, which is the isoform predominantly expressed in the liver and adipose tissues, plays an important role in the synthesis of monounsaturated long-chain FAs from saturated fatty acyl-CoAs. According to Li *et al*.^39^, saturated fatty acids (such as palmitic acid, 16:0) exert lipotoxic activity in liver cells, inducing caspase activity and, therefore, apoptosis. To overcome this, liver cells upregulate the activity of SCD, because MUFAs (such as palmitoleic acid, 16:1) can be stored in cells without the toxic effects observed with saturated fatty acids. The authors suggest that the accumulation of fat in liver is a mechanism of adaptation to avoid liver cells death^39^. Hepatic SCD1 activity, has been shown to correlate with non-alcoholic fatty liver disease (NAFLD)^40^. In the present study, we observed increased 16:1/16:0 ratio in HP diet indicating a higher activity of the SCD1 enzyme, suggesting that, high-fat diet richer in HP –grown in Brazil– contributes to hepatic fat accumulation with potential to promote NAFLD. In fact, Puri and colleagues^41^ have previously performed lipidomic analysis on human healthy livers, and those of steatosis and non-alcoholic steatohepatitis patients. From their data we can obtain the SCD ratio for healthy (0.053), steatosis (0.076) or steatohepatitis (0.062) liver patients, which are within the same range of our findings. Yamada and colleagues^42^ analysed also similar groups and found a clear difference between healthy (0.10) and non-alcoholic steatohepatitis (0.13) patients. They also correlated these data with different histopathological analysis and showed a significant correlation between these ratios and the progression degree of NAFLD^42^. On the other hand, the increased glucose levels observed in the HP group, might be indicative of hyperinsulinemia, and insulin can also upregulate the activity and expression of SCD^43^. Overall, our data suggest that HP diet, by mechanisms still unknown, alters liver function when compared to that of AP supplementation. Whether or not this HP given in a normolipidemic diet also alters liver function, is a subject for future investigations in this experimental model.

In order to identify liver miRNAs that could be modulated by these high-fat diets richer in HP and AP, we evaluated hepatic miRNAs in this experimental model. To the best of our knowledge, no specific studies have been conducted either on the qualitative liver expression of miRNAs, or on their modulation by these hyperlipidic diets. miRNAs are powerful post-translational regulators that mediate gene regulation by providing the means of fine tuning entire gene networks. In this manner, the modulation of a small number of miRNAs allows the regulation of many genes involved in different pathways. The characterization of the miRNAs modulated by the Hybrid palm diet vs. the African palm diet, has allowed us to identify 10 miRNAs whose expression is consistently altered under the HP diet on marmosets of both sexes. Analysing the putative gene targets to these miRNAs and studying the pathways in which these genes are involved by gene ontology, five pathways have risen that could be involved and/or mediating the effects observed on the experimental animals under the HP diet. It is worth to highlight three of these pathways due to their relationship to lipid metabolism: (a) Nicotinic acetylcholine receptor signaling pathway, nicotinic acetylcholine receptors (nAChRs) are known to interact with anti-inflammatory pathways and have been implicated in control of appetite and body weight, as well as lipid and energy metabolism^44^; (b) Cell adhesion molecules (CAMs), adhesion of leukocytes to endothelial cells via cell adhesion molecules is thought to be pivotal in the initiation of atherosclerosis^45^, and it has been described that certain miRNAs play important roles in lipid uptake and adhesion molecule expression in oxLDL-effected macrophages/DCs^46^; and (c) Peroxisome, it is well known the role that peroxisomes play in cellular lipid metabolism, being involved in key pathways such as fatty acid beta-oxidation, etherphospholipid biosynthesis and fatty acid alpha-oxidation^47^. These pathways could be mediating an inflammatory response to the observed hepatic damage (namely, increment of AST/ALT ratio and liver size) in an attempt to recover normal liver function. Interestingly, putative gene targets to some of these miRNAs were also related to glucose metabolism or glucose transport, such as Sirtuin 1 (Sirt1) or Sortilin 1 (Sort1), respectively, and several other insulin signalling/receptor related genes including: Vamp2, Hnf4a, Cacna1c, Stx1a, Cacna1d, Snap25, Grb2, Fgf23, Fgf1, or Igf1r. Future experiments will determine whether the expression levels of the putative target genes are modulated by the HP diet.

Some limitations of the present study should be noted though. For instance, the limited number of subjects has reduced the possibility (if any) to find any differences attributed to sex, given the case that these differences exist. Indeed, sex differences may also influence lipid metabolism^48^. The inability to obtain liver biopsies (due to ethical concerns) at time 0 of the intervention, and the inability to obtain enough biopsied tissue for additional histological analysis, still the gold standard method for diagnosing liver disease despite its limitations^49^, has prevented a more thorough and detailed characterization of the effects induced by the tested diets. The strength of our study relies on the use of marmoset as a non-human primate model and a strict control of the fed diet. Nonhuman primates have a lipid metabolism close to ours and provide a very good model for metabolic disorders^22^. The control of the fed diet reduces the variability inherent to free will subjects, giving strength to the statistical analysis. Moreover, diet is known to change not only classically used biochemical parameters, but also novel biomarkers such as miRNAs^50^.

In conclusion, our study provides evidence that HP supplementation in the diet of the common marmoset can induce liver abnormalities not easily detected by classical biochemical parameters (i.e. plasma lipid levels). Our data suggest that overconsumption of HP should be monitored carefully, as its consumption, compared to that of AP, might rise important concerns related to hepatic metabolism. Further studies will be needed to discern the differences of HP supplementation with the variety used in other previous studies.

## Material and Methods

### Animals

Twenty common marmosets (*Callithrix jacchus*, ten males and ten females) were used in this study. The animals were housed in couples in species-specific cages of stainless steel with internal sliding wall (squeeze-back system), according to the established guidelines in the Guide for the Care and Use for Laboratory Animals^51^.

The animals were fed commercial New World monkey pellets (Megazoo® P25, Betim, MG, Brazil) and water ad libitum, as well as low-fat fruits and vegetables offered daily as environmental enrichment, to break the food monotony, following the recommendations of the National Council for Animal Experimentation Control^52^. Care and ambience followed the environmental enrichment protocol for callitrichidae as specified by the Husbandry Guidelines for Callitrichidae^53^.

The marmoset experiments were approved by the Ethics committee for the use of experimental animals of the Health Care Center of the Federal University of Rio de Janeiro, under reference number INJC014. All experiments were performed in accordance with relevant guidelines and regulations.

### Experimental diets

To perform the study, primates were divided into 2 groups (n = 10:5 males and 5 females) subjected to two different diets: African Palm (AP) and Hybrid Palm (HP) groups. The dietary intervention consisted of a 3-month metabolic insult with hyperlipidic (41% of the energetic value) and normoglicid diet, with a high glycemic index, that differed only in the lipid source of the diet: HP vs. AP. Experimental diets were made by Prag Soluções (São Paulo, BR), following the recommendations of the European Association of Zoos and Aquaria^53^ as showed in Table 1. Palm oils (*E. guineensis* and *E. oleifera* interspecific hybrid) were kindly provided by AGROPALMA (Belém, Pará, Brazil).

### Body weight and biometry

Body mass was measured at each blood collection, once the animals were sedated. Biometrics were also performed at this moment to determine the measure of body length (from occipital protuberance up to the first coccygeal vertebra), tail (from first caudal vertebra to tail end), abdominal perimeter (measured where the umbilical scar is located) and thoracic perimeter (measured around the axillary region) with anthropometric tape (Fig. 1).

### Collection and analysis of biological samples

#### Biochemical and hematological analyzes

At the beginning of the study we characterized the animals running hematological and immunological analysis, as well as determining hepatic and renal status. Also, blood samples were taken before dietary intervention (basal) and after 1, 2 and 3 months within the nutritional interventions (Fig. 1).

#### Blood collection

After 12 hours of fasting, animals were sedated with intramuscular ketamine (10 mg/kg) and blood was collected by puncture in the femoral vein (0.8 to 1.0 mL) and separated in aliquots with different anticoagulants: in tubes containing heparin (20 IU/ml blood) or EDTA (ethylenediaminetetraacetic acid, 1.8 mg/ml blood), as required to run the different intended analyzes. Samples with heparin were sent under refrigeration to Laborlife Clinical Analyzes (Rio de Janeiro) to determine biochemical parameters such as: total cholesterol and its fractions, triglycerides, and glycaemia by means of colorimetric chemical kinetic assays with an automated clinical analyzer (Metrolab 2300). The samples collected in EDTA were immediately centrifuged at 3,000 rpm for 15 minutes at 4 °C. Plasma and resulting erythrocytes were frozen at −80 °C for further plasma lipoprotein analysis by fast protein liquid chromatography (FPLC) and assessment of erythrocyte fatty acid profile by gas chromatography, respectively (Fig. 1).

#### Collection of hepatic tissue

For hepatic biopsy, at the end of the experiment, the animals were fasted for 12 hours and anesthetized with ketamine (7.5 mg/kg) and xylazine (1.5 mg/kg), both intramuscularly. The hepatic tissue was collected by laparotomy wedge biopsy. Samples were immediately frozen in liquid nitrogen and stored at −80 °C for further analysis (Fig. 1).

#### Histological analysis of hepatic tissue

Tissues (defrosted) from animals fed AP or HP were fixed in formalin, paraffin embedded, and sections were stained with hematoxylin and eosin (H&E) and Pico Sirius Red. Images were captured with a camera DP72 coupled in a microscope BX51, both Olympus. Three hepatic biopsies of each group were processed.

### Fatty acid profile of diets and biological samples

The fatty acid profiles of the diets and hepatic tissue were determined by liquid-gas chromatography according to the AOCS Ce2b-11 method^54^, the internal standard C13:0 being added for quantification of the methyl esters.

The lipids of the experimental diets were extracted and purified using the Bligh and Dyer method^55^. The alkaline direct methylation method (Ce 2b-11), described by the American Oil Chemists’ Society^56^, was used for transesterification of the oils extracted from the diets, as well as for the extraction, purification, and transesterification of the liver samples. Tridecanoic acid (13:0) (Sigma Chemical Co.) was added to each methylation tube as internal standard. Fatty acid methyl esters were separated and quantified using the Agilent Technologies 7890A CG System with a flame ionization detector and a 100 m × 0.25 mm capillary column with 0.2 µm inner diameter (Supelco Inc., PA, USA). Hydrogen was used as carrier gas. The fatty acid methyl esters were identified based on purified standards (Nu-Chek Prep. Inc., mix 463), and their quantification was performed as a function of the corresponding peak areas compared to that of the internal standard.

### Liver volume measured by computer tomography (CT)

CT scans were performed on all animals prior to and at the end of the dietary intervention in an Optima PET/CT560 equipment, located at the Nuclear Medicine Service of the Hospital Universitário Clementino Fraga Filho- Rio de Janeiro, UFRJ. The settings for CT scans were 120 kVa, 80 mA and slices of 0.65 mm. The volume of the livers was calculated using OsiriX Lite v.7.0.4 software, where each tomographic slice was manually segmented, and its volume was calculated later. A 3D image of the animal bone structure was processed for a better view of the location of the region of interest.

### Total lipid determination in liver

Total fat in liver samples was determined by the gravimetric method after lipid extraction. Lipid extraction was performed as described by Bligh and Dyer^55^, and the results are expressed as percentage.

### Plasma lipoprotein profile

Equal volumes of plasma from every marmoset in each group were pooled and 220 μl of pooled plasma were subjected to gel filtration by fast protein liquid chromatography (FPLC) using a Superose 6 HR 10/30 column (Pharmacia). The samples were eluted with 150 mM NaCl, 10 mM Tris-HCl, 2 mM Na_2_-EDTA and 0.02% NaN_3_, pH 7.4, at a flow rate of 0.3 ml/min. Fractions of 0.4 ml were collected and cholesterol and triglyceride concentrations in each fraction were determined enzymatically (Centronic GmbH, Wartenberg, Germany).

### miRNA selection

We selected the miRNAs presenting the highest sequence similarity with human miRNAs. Using data related to miRNAs sequence provided by The Marmoset Genome Sequencing and Analysis Consortium (The Common Marmoset Genome Provides Insight into Primate Biology and Evolution^57^), we compared the marmoset miRNA sequences with the assessed human miRNAs, including in our screening the sequences presenting 100% similarity with human miRNAs, and sequences with up to two mismatches outside the seed sequence (nucleotides 2–8) and/or differing in up to two nucleotides in length. The number of marmoset miRNAs selected was 370, to which we added miRNAs to be used as normalizing miRNAs.

### RNA extraction and miRNA screening

Liver total RNA from biopsied tissue was isolated using Qiazol Reagent and miRNeasy Mini kit columns (Qiagen). RNA was quantified using a NanoDrop-1000 Spectrophotometer and purity was determined by calculating the 260/280 nm and 260/230 nm ratios. RNA integrity was assessed using the Agilent 2100 Bioanalyzer. For the selected (see above) miRNA screening, cDNA was synthetized from total RNA (1000 ng) using miScript® II RT Kit (Qiagen), following the manufacturer’s protocol. miRNA quantification was performed by real time PCR (qRT-PCR) using the miScript SYBR®Green qPCR Master Mix (Qiagen) on a 7900HT fast real time PCR system (Applied Biosystems) using a 384-well plate format. Reactions were performed in a final volume of 10 µl following these cycling conditions: 15 min at 95 °C for one cycle, then 40 cycles at 94 °C for 15 s and 58 °C for 30 s. The dissociation curve was analyzed at 95 °C for 15 s, followed by one cycle with increasing temperature starting at 60 °C and ending at 95 °C. RNU6 and RNU1A1 were used as reference small RNAs for normalization. miRNA relative expression analysis was performed using the GenEx software (MultiD Analyses AB, Sweden).

### miRNA validation

Validation of selected miRNAs from the primary screening were performed by Real-time RT PCR using a second different pair of primers. Reactions were performed using cDNA obtained as indicated previously in 384-well plates, using miScript SYBR green Mastermix (Qiagen) and gene expression determined using a 7900HT Fast Real-time PCR System (Applied Biosystems) as described above. Specific oligos for Callitrix miRNAs were used and values were normalized by the housekeeping small RNAs RNU1A1 and RNU6.

### Marmoset miRNA target prediction

Marmoset transcripts 3′UTR sequences were obtained from Ensemble database (Dataset C_jacchus3.2.1), ending up with a set of 30642 sequences. After the selection of differentially expressed microRNAs, putative targets for these miRNAs were determined using the miRanda^58^ algorithm v.3.3, setting a minimum alignment score of 150 as a threshold for a valid mRNA-miRNA interaction. For functional analysis of these predicted targets, marmoset genes were mapped to their corresponding human orthologous, and enriched pathways and gene ontology (GO) biological processes were identified using the Genecodis enrichement analysis tool^59^.

### Statistical analysis

Results are expressed as mean ± error standard of the mean (EPM). Kolmogorov-Smirnov test was used to test the normality of the data. Those data that were not parametric were log transformed. *t* test was performed to establish statistical significances between experimental groups with GraphPad Prism program, version 7. The level of significance was set at 5% (p < 0.05).

## Electronic supplementary material

Supplementary Information
