# Supplementary material for: Dietary supplementation with hybrid palm oil alters liver function in the common Marmoset
Source: Sci Rep. 2018 Feb 9;8:2765. doi: 10.1038/s41598-018-21151-0 (PMC5807430; doi:10.1038/s41598-018-21151-0)

Dietary supplementation with hybrid palm oil alters liver function in the common Marmoset

Flavia Spreafico[1,3*], Rafael Carvalho Sales[1], Judit Gil-Zamorano[2], Priscylla da Costa Medeiros[3], Maria-Jesús Latasa[2], Monique Ribeiro Lima[1]; Sergio Augusto Lopes de Souza[3], Roberto Martin-Hernández[2], Diego Gómez-Coronado[4,5], Eduardo Iglesias-Gutierrez[6,7], Diana C. Mantilla-Escalante[2], Maria das Graças Tavares do Carmo[1], Alberto Dávalos[2*].

[1]Instituto de Nutrição Josué de Castro, Universidade Federal do Rio de Janeiro, Rio de Janeiro, Brazil.

[2]Laboratory of Epigenetics of Lipid Metabolism, Instituto Madrileño de Estudios Avanzados (IMDEA)-Alimentación, CEI UAM+CSIC, Madrid 28049, Spain.

[3]Laboratório de Marcação de Células e Moléculas, Departamento de Radiologia, Faculdade de Medicina, Hospital Universitário Clementino Fraga Filho, UFRJ, Rio de Janeiro, Brazil.

[4]Servicio de Bioquímica-Investigación Hospital Universitario Ramón y Cajal, IRYCIS, Madrid 28034, Spain.

[5]CIBER Fisiopatología de la Obesidad y Nutrición (CIBEROBN), Instituto de Salud Carlos III, Spain.

[6]Department of Functional Biology (Physiology), University of Oviedo, Oviedo 33003, Spain.

[7]Universidad Autónoma de Chile, Santiago 7500912, Chile.

*Correspondance to: (F. Spreafico) flaviasfer@gmail.com or (A. Dávalos) alberto.davalos@imdea.org

Supplementary Information

Table S1. GO analysis of `predicted genes modulated by hybrid palm oil-modulated miRNAs

Supplementary Figure S1: Body weight progression (in grams) of the experimental groups during the nutritional intervention expressed as the mean of body weight ± standard error, (n=10 animals per group). No significant differences were found between the groups according to t test at $p<0.05$. AP: African Palm Group; HP: Hybrid Palm Group.

Supplementary Figure S2: Lipoprotein cholesterol and triglyceride profiles during dietary supplementation. (n=9 animals per group). AP: African Palm Group; HP: Hybrid Palm Group. A: AP group at 0, 1 and 3 months of dietary treatment, respectively. B: HP group at 0, 1 and 3 months of dietary treatment, respectively. VLDL: very low-density lipoproteins; LDL: low density lipoproteins; HDL: high density lipoproteins.

Supplementary Figure S3. Liver miRNAs validation by RT-qPCR. Hepatic miRNAs were analysed by RT-qPCR in both sexes. AP: African Palm Group; HP: Hybrid Palm Group. Values are expressed as mean ± standard error (n=9 animals per group).

Table S1. GO analysis of `predicted genes modulated by hybrid palm oil-modulated miRNAs

ID	Description	Adjusted *P* value	Nº of genes	Genes	microRNA's
Panther: P00044	Nicotinic acetylcholine receptor signaling pathway	0.00885829	18	Cacnb1, Chrng, Myh11, Myo5c, Stx1a, Vamp1, Cacna1c, Cacna1d, Chrnb3, Myo18a, Myo3a, Acta1, Snap25, Actc1, Actr1b, Chrnb2, Vamp2, Vamp8	calJac-let7b-5p, calJac-miR17b-5p-1, calJac-miR25b-3p, calJac-miR484-1, calJac-miR488-3p1, calJac-miR592, calJac-miR877-5p
Kegg: 04514	Cell adhesion molecules (CAMs)	0.000000917	24	Alcam, Cd8b, Cldn16, Cldn18, Itgb8, Sdc2, Vcan, Cd4, Cldn11, Cldn15, Cldn19, Hla-Doa, Itga9, Itgb7, Ncam1, Sdc1, Cd8a, Cdh3, Itgav, Madcam1, Mpzl1, Ocln, Sell, Siglec1	calJac-miR17b-5p-1, calJac-miR25b-3p, calJac-miR4745-5p, calJac-miR484-1, calJac-miR488-3p-1, calJac-miR877-5p
Kegg: 04146	Peroxisome	0.0000909	12	Acsl4, Acsl6, Crot, Pecr, Pex10, Gstk1, Hacl1, Hsd17b4, Paox, Pex11b, Pex26, Pex5	calJac-let7b-5p, calJac-miR17b-5p-1, calJac-miR339-3p, calJac-miR488-3p-1
GO: 0031175	neuron projection development	0.00140149	10	Cdk5, Cdk5r1, Cntn2, Igf1r, Lamb1, Lingo1, Tbc1d24, Cdh1, Lamc1, Rb1	calJac-let7b-5p, calJac-miR17b-5p-1, calJac-miR484-1, calJac-miR488-3p-1, calJac-miR592, calJac-miR877-5p
GO: 0030154	cell differentiation	0.0662095	109	Cbfa2t3, Clic4, Cplx2, Dusp6, Fgf23, Gnptab, Igsf9, Nme1, Ptpru, Rasgrp1, Robo2, Ascl2, Bmpr1b, Cadm1, Dclk1, Dll1, Dll4, Dmrt1, Ereg, Fcrla, Kif2a, L1cam, Mapk7, Pappa, Pkdcc, Racgap1, Rnf17, Robo1, Rufy3, Sema3a, Sema3d, Sh2d2a, Siah1, Srpk1, Stmn1, Ttll7, Usp42, Abhd5, Angpt2, Arhgap24, Bmp1, Bzw2, Camk2g, Caprin1, Caprin2, Col19a1, Csf1, Fgf1, Ggnbp2, Gldn, Grb2, If1a, Ift81, Jmjd6, Mgp, Mkl2, Nfatc4, Piwil2, Sema4b, Sema4g, Sema6d, Sort1, Sqstm1, Strbp, Suv39h2, Utp14c, Acsbg2, Chl1, Osr1, Clptm1, Dbn1, Fev, Hnf4a, Huwe1, Ndel1, Ntng1, Ppard, Slc9a1, Smurf1, Spata20, Tnfrsf12a, Asz1, Bmpr1a, Chrdl1, Cit, Dmrt3, Epas1, Gna12,	calJac-let7b-5p, calJac-miR17b-5p-1, calJac-miR25b-3p, calJac-miR339-3p, calJac-miR4745-5p, calJac-miR484-1, calJac-miR488-3p-1, calJac-miR592, calJac-miR877-5p

				Myt1l, Naa15, Nhs, Nrp2, Pafah1b1, Pax5, Pax6, Rbm38, Rnf114, Slc26a8, Tnfsf11, Tp53, Dcx, Dhcr7, Dmrtc2, Hand2, Ngef, Paqr5, Sirt1, Tgfb1i1, Vtn	

Supplementary Figure S1

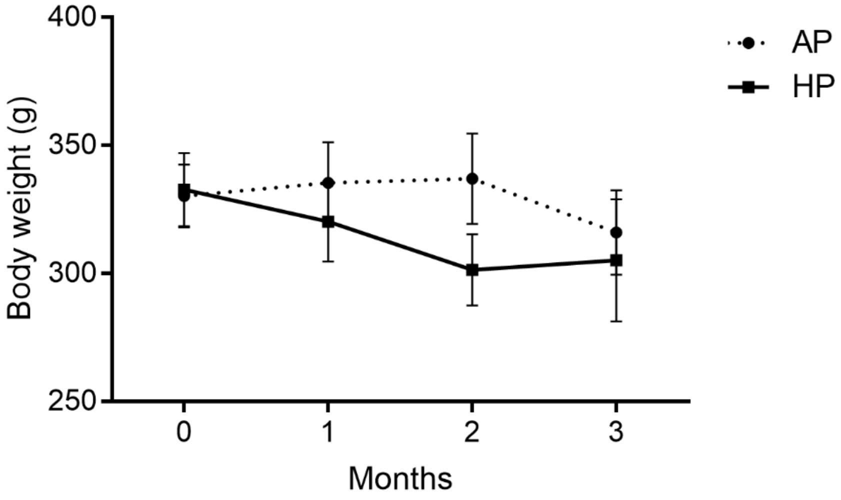

Supplementary Figure S2

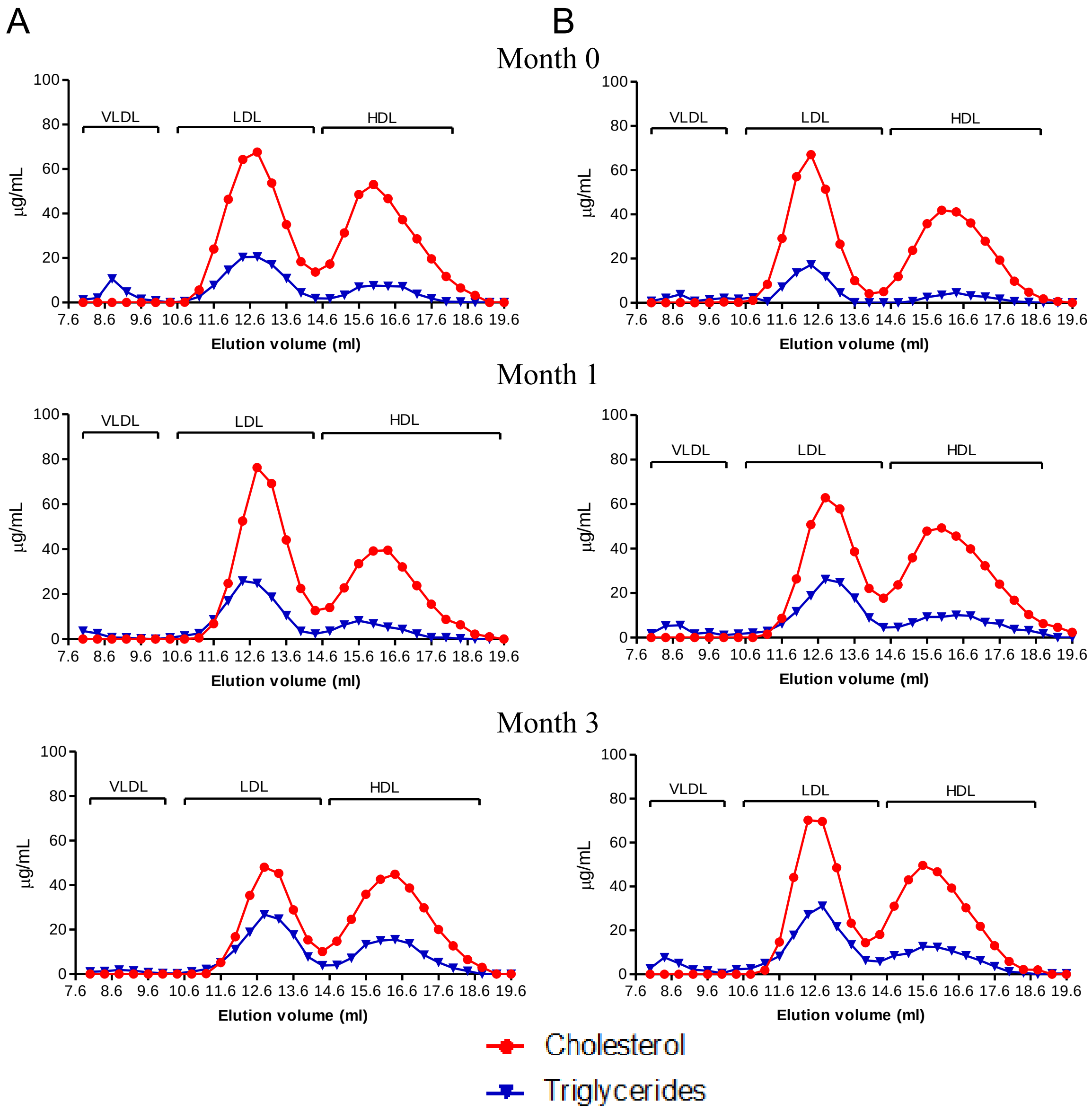

Supplementary Figure S3

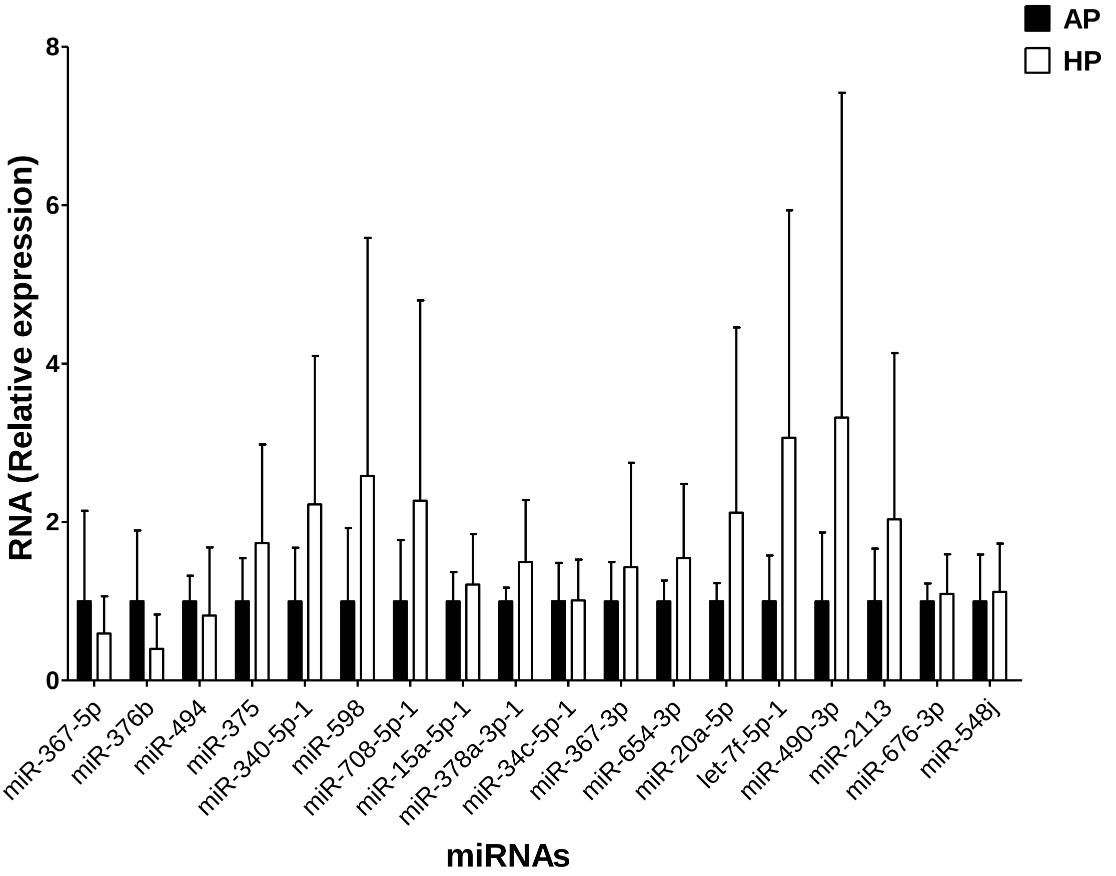

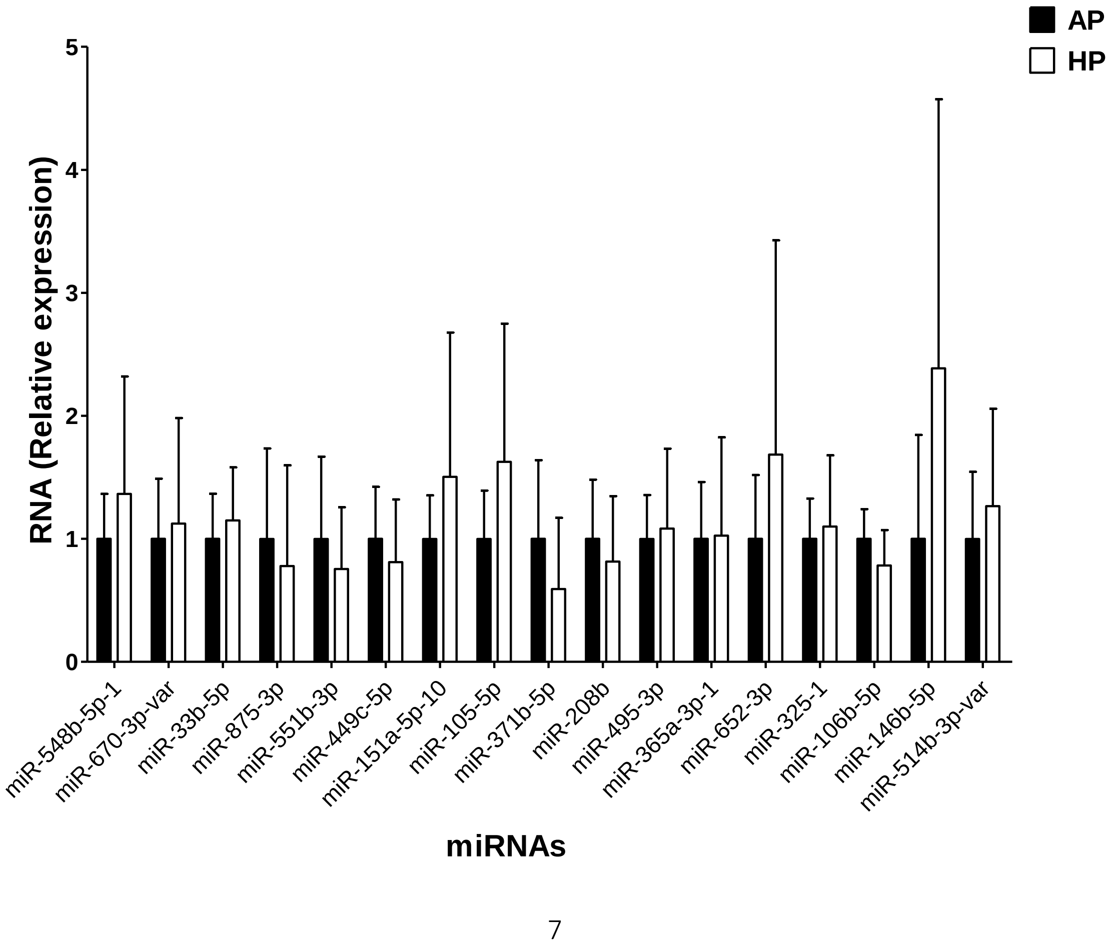

Supplement: Supplementary file 1 — Supplementary Information [file 41598_2018_21151_MOESM1_ESM.pdf]
